# Early Childhood Oral Health: Insights into Knowledge, Preventive Practices, and Risk Awareness from a Croatian Cross-Sectional Study

**DOI:** 10.3390/pediatric17060130

**Published:** 2025-12-03

**Authors:** Marija Matijević, Marija Badrov, Lidia Gavić, Antonija Tadin

**Affiliations:** 1 Department of Restorative Dental Medicine and Endodontics, Study of Dental Medicine, School of Medicine, University of Split, 21000 Split, Croatia; marijaa.matijevic@gmail.com (M.M.); marija.badrov@mefst.hr (M.B.); atadin@mefst.hr (A.T.); 2 Department of Dental Medicine, University Hospital of Split, 21000 Split, Croatia

**Keywords:** children, early childhood caries, Croatia, oral health, oral hygiene, parental knowledge

## Abstract

*Aim:* Early childhood caries (ECC) is a widespread and multifactorial oral disease that affects children globally. Parents’ knowledge, attitudes, and behaviors are crucial in preventing ECC and supporting oral health. This study evaluated Croatian parents’ understanding of children’s oral health, their awareness of ECC risk factors, and their oral hygiene practices. *Materials and methods:* A cross-sectional study was conducted using an anonymous and voluntary online questionnaire from October to December 2024 among 948 parents of children aged 1–7 years across Croatia. The study assessed parents’ knowledge of oral health, their understanding of the relationship between risk factors and early childhood caries, habits related to oral hygiene care, children’s experiences with oral health problems, parents’ self-assessment of their knowledge, as well as both their own and their children’s general and oral health and hygiene practices. Data were analyzed using descriptive statistics, Chi-square test, Mann–Whitney U test, and Kruskal–Wallis test. *Results:* Overall parental knowledge was moderate, with significantly higher scores among older parents, those with university education, healthcare workers, and families with higher incomes (*p* < 0.05). Parents demonstrated good awareness of the importance of supervising tooth brushing until age seven (93.8%) and fluoride use (81.8%); yet gaps persisted regarding bacterial transmission, tooth eruption, and early orthodontic evaluation. Preventive dental visits were frequently delayed, and only 25.0% of parents reported using interdental cleaning aids. Caries was the most common oral health issue among children (22.3%). *Conclusions:* Despite moderate awareness and some adherence to preventive measures, significant knowledge and practice gaps remain among Croatian parents. Targeted educational interventions and nationwide preventive strategies are necessary to strengthen oral health literacy and reduce ECC prevalence.

## 1. Introduction

Dental caries is one of the most common diseases in the world, affecting all socioeconomic groups. It is defined as a bacterially induced, chronic, and irreversible disease of multifactorial etiology, causing progressive demineralization and loss of enamel and dentin [1,2]. Early childhood caries (ECC) involves the presence of caries or fillings on one or more primary, deciduous teeth and affects children up to approximately six years of age [3]. Cariogenic bacteria are transmitted mostly from mother to child by kissing or sharing eating utensils, a process known as vertical transmission. The most common causative agent of caries is the bacterium *Streptococcus mutans* [4]. The World Health Organization estimates that dental caries affects approximately 50% of children with primary dentition worldwide [3,5].

Primary dentition begins with the eruption of the lower central incisors at around six months and is typically complete by the age of three, with all 20 teeth [6]. Although temporary, primary teeth play a key role in speech development, mastication, facial growth, space preservation for permanent teeth, and psychosocial well-being [7]. The enamel of primary teeth is thinner and less mineralized, which makes primary teeth more susceptible to the development of caries [8]. The development of ECC is also influenced by a diet rich in added sugars, a lack of the child’s cooperation in maintaining oral hygiene, parents’ lack of knowledge about oral health, and a delayed first dental visit [9]. Timely implementation of preventive measures ensures long-term oral health, including healthy teeth and a healthy overall oral cavity [10]. Preventing oral problems in children involves proper oral hygiene, a balanced diet, regular dental visits, avoiding harmful habits, and active parental involvement and education [11]. Preschool children adopt behavioral patterns from their parents, which makes parental knowledge, attitudes, and habits crucial [12]. Bacterial biofilm can only be removed mechanically; therefore, parents should assist or supervise tooth brushing until the child is at least seven years old. Tooth brushing should begin with the eruption of the first primary tooth, twice daily for two minutes, using fluoridated toothpaste—a smear for children under three and a pea-sized amount for those over three [13]. Fluoride is considered one of the most important and effective preventive agents. Regular topical use in low doses promotes remineralization and incorporates fluorapatite crystals into the tooth enamel, making it more resistant to acid and demineralization [14]. The first dental visit should ideally take place after the eruption of the first primary tooth and before the child’s first birthday [15]. Early dental visits enable the timely detection and management of potential anomalies and oral health issues, while also providing parents with guidance on prevention and oral hygiene. Delaying visits until problems occur often leads to painful interventions and increases the risk of dental anxiety and phobia in children [16]. The eruption of the first permanent molars usually marks the beginning of permanent dentition and represents the optimal time for the first orthodontic evaluation [17].

Parent–child interaction plays a crucial role in shaping a child’s attitude toward dental care. Recent evidence suggests that parental dental anxiety and fear of treatment can be directly transmitted to children, influencing their perception of dental visits and overall cooperation during procedures. Moreover, parents’ emotional responses to dental trauma or pain often determine how promptly they seek professional help for their child. When parents display calmness, trust, and positive communication regarding dental treatment, children are more likely to develop adaptive coping mechanisms, reduced anxiety, and long-term positive oral health behaviors [18]. Recent Croatian and European studies show that parental dental fear and anxiety are strongly associated with dental anxiety and avoidance behaviors in children, with psychosocial mechanisms such as modeling, family milieu, and cognitive vulnerability mediating this transmission. Parental anxiety, negative dental experiences, and psychological stress are significant predictors of both child dental anxiety and poorer oral health outcomes, including increased risk of caries and reduced dental self-efficacy; these effects are mediated by intra-family relationships, parental attitudes, and the transmission of threat perceptions from parent to child [19,20,21,22].

Previous studies on children’s oral health in Croatia have revealed numerous challenges and the need for improved preventive strategies. A 2015 study comparing the trends of DMFT index (Decayed, Missing, Filled Teeth Index) in Croatia and Europe showed a DMFT index of 4.14 among six-year-olds in Croatia [23]. Similarly, a 2019 study concluded higher caries prevalence among children from rural areas than their urban peers, while another study conducted the same year among school-age children on Croatian islands showed a strong influence of dietary and hygiene habits on oral health [24,25]. Results of studies from 2022 and 2024 indicated a connection between parental factors—especially dental anxiety and lack of knowledge—and the development of dental anxiety in children [26,27]. Additionally, a 2025 study among parents of preschool children showed that insufficient knowledge about nutrition and breastfeeding increases ECC risk [28]. Children from vulnerable groups, such as those from SOS Children’s Villages, have shown lower levels of oral health compared to their peers from the general population, while studies from 2024 and 2025 among children with special needs highlighted reduced oral health-related quality of life and additional challenges faced by their caregivers [29,30,31]. Although some parts of Croatia, such as Primorje-Gorski Kotar County, have already implemented oral health promotion models, most data derive from regional studies [32].

Croatia still lacks nationally representative and longitudinal data on children’s oral health. Few intervention studies assess specific preventive programs, and vulnerable groups—such as children with special needs, those in alternative care, or migrant communities—are under-researched. The role of digital media and apps in promoting oral health, as well as the impact of socioeconomic and cultural factors, remains neglected. As primary caregivers and role models, parents need up-to-date knowledge of oral health and hygiene, as well as its proper application, to effectively care for their children’s oral health. This study aims to assess parents’ knowledge about oral health and ECC risk factors and to analyze their attitudes and habits regarding children’s oral hygiene. Providing insight into possible gaps in knowledge and habits will enable us to improve and implement programs promoting children’s oral health and parental education. The hypothesis is that parental knowledge and practices are insufficient and inadequate.

## 2. Materials and Methods

### 2.1. Study Design and Participants

This cross-sectional study was conducted by the Department of Restorative Dentistry and Endodontics at the Faculty of Medicine, University of Split, during the period from October to December 2024. It was approved on 7 June 2024, by the Ethics Committee of the School of Medicine, University of Split (Class: 029-01/24-02/0001; Reg. no.: 2181-198-03-04-24-0066). The study followed the guidelines of the Checklist for Reporting Results of Internet E-Surveys (CHERRIES) [33]. The study also implemented measures to prevent duplicate entries by restricting one response per IP address. Incomplete surveys were excluded from the analysis, and the overall response rate was calculated based on the number of valid submissions, ensuring the reliability and accuracy of the collected data.

Data were collected using a non-probability convenience sampling method through an online questionnaire created with Google Forms. The questionnaire was distributed to parents of children aged one to seven years, encompassing early childhood and preschool age, via social networks, and online parenting groups. This approach aimed to ensure a geographically diverse sample of respondents from various regions of Croatia. Participants were also asked to forward the questionnaire to their acquaintances using a snowball sampling technique. At the beginning of the questionnaire, all respondents were thoroughly informed about the purpose of the study, the number of questions, the estimated time required to complete the survey, the methods of data storage and use, the identity of the researchers, as well as the anonymity and confidentiality of the data, which would be used exclusively for scientific research. On the first page of the questionnaire, respondents provided their voluntary and informed consent to participate in the study.

Inclusion criteria for the study encompassed the adult general population of the Republic of Croatia: parents of early childhood and preschool-aged children up to seven years old, who voluntarily agreed to participate and completed the online questionnaire in full. Exclusion criteria included parents of children older than seven years and respondents who submitted incomplete or inadequately filled questionnaires. According to the Croatian Bureau of Statistics data from 2023, the estimated number of families in the Republic of Croatia with children under seven years old was approximately 150,000 [34]. A total of 949 participants took part in this study. The minimum required sample size for the study’s validity (N = 377) was calculated using the Sample Size Calculator software (Inc. RaoSoft^®^, Seattle, WA, USA), with a 95% confidence interval and a 5% margin of error [35].

### 2.2. Survey

The questionnaire was developed in Croatian by a specialist in endodontics and restorative dental medicine and a sixth-year dental student. It was designed and adapted based on previously conducted studies on similar topics, which were published on the PubMed platform [36,37,38]. Before the main study, a pilot test was carried out on a sample of 15 parents who met the inclusion criteria. These participants confirmed the usability and functionality of the questionnaire. The estimated time required to complete the survey was approximately 15 min. The questionnaire was revised based on the feedback and suggestions for improvement provided by the pilot participants. Therefore, their responses were not included in the main study sample.

The questionnaire consisted of 77 questions divided into six sections. The initial section included 11 questions related to the sociodemographic characteristics of the respondents (parents and their children). These questions covered the parents’ age and gender, educational level, employment status, family socioeconomic status, place of residence, employment in healthcare, number of children in the family, as well as the child’s age, gender, and the timing of the eruption of their first primary tooth. The second section assessed knowledge about children’s oral health through 12 questions with “True” and “False” answer options. This section had a scoring system awarding 1 point for each correct answer and 0 points for incorrect answers. The total score for each respondent was calculated based on the number of correct answers, with a maximum possible score of 12 points. According to Bloom’s cutoff criteria, participants’ overall knowledge was classified as excellent for scores between 80% and 100% (9.6–12.0 points), moderate for scores between 60% and 79% (7.2–9.5 points), and poor for scores below 60% (<7.2 points) [39]. The next section comprised 20 questions addressing the respondents’ knowledge of risk factors associated with early childhood caries. Dichotomous “Yes” and “No” options were provided, with “Yes” being the correct answer. Respondents received one point for each correct answer (“Yes”) and zero points for incorrect answers (“No”) with a maximum score of 20 points. Using Bloom’s categorization based on the same percentage thresholds, excellent knowledge was defined as a score between 16.0 and 20.0 points (80–100%), moderate knowledge as 12.0 to 15.8 points (60–79%), and poor knowledge as 0.0 to 11.9 points (below 60%). The fourth section consisted of 13 dichotomous questions related to respondents’ habits in caring for their child’s oral health, with “Yes” and “No” answer options. Topics included frequency and duration of tooth brushing, use of fluoride toothpaste, separate toothbrushes for parent and child, replacing the toothbrush every three months, assisting the child during brushing, dental visits, and similar behaviors. The fifth section comprised 14 questions investigating the children’s experiences with oral health issues such as caries, gingivitis, dental trauma, bruxism, malocclusion, and related conditions. The final section included 7 questions focused on respondents’ self-assessment of various health aspects, including their general health, oral health, knowledge of oral health, personal oral hygiene practices, as well as their child’s general health, oral health, and oral hygiene routines. Responses were given on a scale with options: “Poor,” “Good,” and “Excellent.”

### 2.3. Data Analysis

The primary outcome of this study was to gain insight into parents’ knowledge of oral health and the factors contributing to early childhood caries, and to relate this knowledge to parents’ demographic characteristics, oral hygiene habits, and the child’s oral status. Continuous variables were presented as means (standard deviations), while categorical variables were expressed as absolute numbers and percentages. The normality of distribution for continuous variables was tested using the Shapiro–Wilk test. To analyze the relationships between parental knowledge and sociodemographic and professional factors, the Mann–Whitney U test and Kruskal–Wallis test were employed. Statistical significance was set at *p* < 0.05. Data analysis was performed using the Statistical Package for the Social Sciences, version 26.0 (SPSS, IBM Corp., Armonk, NY, USA).

## 3. Results

The sociodemographic data of the respondents are presented in Table 1. The study included 948 respondents, of whom 97.2% were women (N = 921). The average age of the parents was 34.3 ± 5.2 years (maximum age = 49, minimum age = 20), and they had an average of 1.7 ± 0.9 children (maximum number of children = 9, minimum number of children = 1). Mothers showed a statistically significantly higher level of knowledge compared to fathers, while parents employed in the healthcare field, although a minority (17.5%, N = 166), had a significantly higher level of knowledge about oral health and risk factors. A university degree was held by 69.8% of respondents (N = 662), and 87.8% (N = 832) of parents were employed. Most parents (74.5%, N = 706) described themselves as average in socioeconomic status, and 73.4% (N = 696) lived in an urban environment. A higher level of education and socioeconomic status correlated with a higher level of knowledge. The average oral health knowledge score was 8.3 ± 2.4 (maximum = 12, minimum = 0, median = 9, Q1 = 7, Q3 = 10), while the knowledge score on the association of risk factors with the occurrence of early childhood caries was 14.1 ± 4.2 (maximum = 20, minimum = 0, median = 15, Q1 = 12, Q3 = 17).

Table 2 presents the characteristics of the respondents’ children. The average age of the children was 3.2 ± 1.7 years (maximum age = 7, minimum age = 1). Parents of nursery-age children (0–3 years) had a significantly higher level of knowledge regarding the association of risk factors with the occurrence of early childhood caries. The number of male and female children was approximately equal, with girls slightly predominating at 51.3% (N = 486). The average age at eruption of the first primary tooth in a child was 6.6 ± 2.2 months (maximum = 17, minimum = 2), and the highest knowledge was demonstrated by parents of children who had the expected onset of tooth eruption, between 6 and 8 months of age.

Table 3 presents the results of the oral health knowledge assessment. The average total knowledge score was 8.3 ± 2.4 out of a possible 12 points. The highest percentage of correct answers, 93.8% (N = 889), was given for the statement: “Parents should at least supervise and assist the child with tooth brushing until the age of seven.” Only 28.9% (N = 274) of respondents knew that the mother’s oral flora is transmitted to the newborn and that a higher presence of cariogenic bacteria in the mother increases the chances of the child developing caries. Poor knowledge was also demonstrated regarding the sequence of eruption of permanent teeth, where only 40% (N = 379) knew that the first permanent tooth in the oral cavity is the first permanent molar, which erupts between the ages of six and seven years. It is important to note that as many as fourteen respondents (1.48%) did not know the correct answer to any of the statements presented, while a commendable score of 12 points, with all correct answers, was achieved by 62 respondents (6.54%).

Table 4 shows the frequency of correct and incorrect answers given by respondents regarding the association of risk factors with early childhood caries. The average knowledge score was 14.15 points with a standard deviation of 4.29 points (maximum = 20, minimum = 0). Seventy-three respondents (7.7%) answered all statements correctly, while 18 respondents (1.9%) did not know the correct answer to any question. Regarding specific statements, 92.7% (N = 879) of parents knew that the consumption of sugary drinks and food before bedtime promotes the development of early childhood caries. Interestingly, only 35% (N = 332) of respondents believed that the social status of parents is related to the occurrence of early childhood caries.

Table 5 presents parental habits regarding the care of their child’s oral health. A habit adhered to by almost all parents (98.8%, N = 937) is the use of separate toothbrushes for the parent and the child. Oral hygiene habits practiced by most parents (over 90%) in caring for their child’s oral health include visits to the dentist if the child has dental caries (93.9%, N = 890) and supervision and assistance with tooth brushing (93.1%, N = 883). The use of dental floss is a habit followed by only 25.1% of respondents.

Table 6 presents the frequency of oral health problems in the respondents’ children. Dental caries was the most experienced disease, affecting 211 children (22.3%), followed by bruxism, which affected 147 children (15.5%). Other problems were relatively rare, with the lowest frequencies observed for gingival infection (2%, N = 19), lip biting (2.3%, N = 22), and oral candidiasis (2.5%, N = 24).

The assessment of parents’ own general and oral health, as well as that of their children, and their hygiene habits, is presented in Table 7. Most parents described their own general and oral health as “Good,” as well as their self-assessment of knowledge about oral health and the performance of oral hygiene. Interestingly, the smallest number of respondents described their oral health, knowledge, and habits as “Poor,” yet they had a higher level of knowledge than those who rated themselves as “Good” or “Excellent.” Sixty-five (6.9%) parents rated their own oral health as “Poor,” but had a statistically significantly better level of knowledge than other parents. They rated their children more positively, with most respondents describing their child’s general and oral health as “Excellent.” However, the oral hygiene of their children was rated slightly lower by 483 (50.9%) respondents as “Good.” Notably, parents of children with poor general health had a higher level of knowledge about oral health.

## 4. Discussion

Parents are responsible for their children’s oral health and pass on hygiene habits through their own example and instruction [40]. Accordingly, this study aimed to assess the level of knowledge of parents of young children, i.e., preschool-aged children, regarding children’s oral health and the risk factors for early childhood caries, as well as to gain insight into their habits related to providing oral hygiene care for their children. The overall knowledge of all respondents about children’s oral health and about the association of risk factors with early childhood caries in both sets of questions was found to be moderate, confirming the hypothesis of this study that parents have insufficient levels of knowledge. In line with this insufficient knowledge, the hypothesis that parents do not provide proper and regular oral hygiene care for their children was also confirmed. This is reflected, among other things, in the fact that only one-third of respondents took their child to the first dental check-up before the age of one, and only 65.3% of children brush their teeth for at least two minutes. Previous studies have confirmed that education improves the knowledge of both parents and children and that children’s oral health and oral hygiene habits are associated with parents’ oral health literacy [27,41].

The results of the study showed that mothers had a significantly higher level of knowledge about children’s oral health and the association of risk factors with the occurrence of early childhood caries compared to fathers, which is consistent with a study conducted in Saudi Arabia, in which mothers demonstrated higher levels of knowledge, while fathers believed that the oral cavity does not need to be cleaned before the eruption of primary teeth and that children can adequately brush their teeth without parental assistance [36]. This outcome can be explained by the social norm that the mother is the child’s primary caregiver and is therefore more involved in general, as well as in dental care, dental visits, and, accordingly, education [42]. Older age, higher education level, and higher socioeconomic status were also associated with higher knowledge among parents in both sets of questions—knowledge about oral health and knowledge about risk factors related to the development of early childhood caries. The Saudi Arabian study reached the same conclusions: older parents had significantly more correct answers than other age groups for all questions, and parents with a university degree had more correct answers compared to parents of other educational levels [17]. An interesting finding emerged from a Polish study, which, despite better knowledge among more educated parents, showed that mothers used less fluoride toothpaste the higher their education level was. The same study confirmed that lower socioeconomic status is proportional to lower parental knowledge [40]. Parents working in healthcare showed a statistically significantly higher level of knowledge, which is unsurprising given that healthcare professionals are expected to be educated about oral health and to take its importance more seriously. Similar results were found in Croatian studies on oral health knowledge among students, where those enrolled in health- and medical-related studies had better knowledge compared to students in other fields [43].

The question for which parents gave the highest number of correct answers regarding children’s oral health—93.8%—was the statement: “Parents should at least supervise and assist their child with tooth brushing until the age of seven.” Excellent knowledge was also demonstrated by 81.8% of parents correctly identifying that fluoride protects teeth from caries, which is almost identical to the 81.5% reported in a U.S. study conducted among the Native American population [37]. Another strong result was for the statement that children’s teeth should be brushed twice daily, with 90.1% of parents answering correctly, compared to 95.2% in the U.S. study. Contrasting results were observed for the question about supervising a child’s tooth brushing until the age of 6–7 years: 93.8% of respondents in this study answered correctly, whereas only 2.1% of parents did so in the U.S. study. A concerningly low percentage of parents (28.9%) were aware that a mother’s oral microbiota is transmitted to the newborn and that a higher presence of cariogenic bacteria in the mother increases the likelihood of caries development. Similarly, in Iraq, 36% of parents correctly recognized that caries is caused by bacteria transmitted to the child via saliva [38]. Additionally, 60% of parents did not know that the first permanent teeth to erupt are the first permanent molars, typically emerging between ages 6 and 7. Furthermore, 58.1% were unaware that children should ideally have their first orthodontic examination around the age of seven. For comparison, 68.8% of orthodontic patients in Saudi Arabia were similarly unaware of the appropriate timing for the first examination [44].

Regarding the association of risk factors with the occurrence of early childhood caries, less than half of parents (45%) believed that active dental caries in the mother/primary caregiver poses a risk for the development of caries in the child. This represents poorer knowledge compared to a South African study, where 60.4% of parents were aware that a mother can transmit bacteria from her oral cavity to the child [45]. Respondents did not recognize a link between early childhood caries and the parents’ social status (35.0%), environmental factors (47.9%), or financial constraints (41.5%), even though research indicates otherwise. For example, a study conducted in the United States on socioeconomic factors associated with the risk and prevalence of dental caries in children found that the highest prevalence of caries occurs in households with the lowest income [46]. Parents demonstrated better knowledge regarding the relationship between dietary habits and caries; 92.7% of parents knew that consuming sugary drinks and foods before bedtime is harmful to teeth, and 91.5% were aware that consuming sugary drinks and snacks more than three times per day between meals is detrimental. These results are considerably better than those reported among Native American populations in the U.S., where only 52.7% of parents considered it harmful for a child to eat before bedtime after brushing their teeth [37].

To initiate preventive measures as early as possible and to detect anomalies promptly, guidelines recommend the first dental visit by the child’s 12th month. However, only 33.3% of participants in this study adhered to this recommendation. Non-compliance with these guidelines is not only a local issue; a study in the United States reported a median age of first dental visit of 7.9 years [47]. The oral hygiene practices most followed by parents were visiting the dentist if the child had dental caries (93.9%) and using separate toothbrushes for the parent and child (98.8%). However, only one quarter of parents used interdental cleaning aids. In a 2025 study conducted among Croatian children on the autism spectrum, only 14% used interdental brushes or dental floss [31]. Fluoride in toothpaste strengthens teeth by making them more resistant to demineralization, incorporating into enamel as fluorapatite. Nevertheless, only slightly more than two-thirds of participants in this study used fluoride toothpaste. A lower prevalence was reported in a study conducted on the island of Korčula among school-aged children, where approximately 50% of participants used fluoride toothpaste [25].

The most common oral health problem in children was dental caries, reported by approximately one quarter of participants, which is consistent with global trends. A study from the United States estimates that 23% of children aged 2 to 5 years have caries in their primary teeth [48]. The DMFT/dmft index among six-year-olds in Croatia reaches as high as 4.14 [23]. A study conducted in Primorje-Gorski Kotar County concluded that girls have a significantly lower DMFT/dmft index than boys, both in primary and permanent dentition [49]. Bruxism, halitosis, and dental trauma were also among the more frequently reported oral health problems in this study.

A growing body of evidence highlights the importance of psychosocial factors in children’s oral health. In particular, parent–child interaction has emerged as a key determinant of how children perceive and respond to dental experiences. Parental dental anxiety, fear of treatment, and emotional reactions to dental trauma can be transmitted to children through modeling and communication, influencing their cooperation and fear responses during dental visits. Such patterns may also contribute to delays in seeking care or avoidance of preventive measures. Conversely, when parents demonstrate calmness, confidence, and a positive attitude toward dental treatment, children are more likely to develop adaptive coping strategies and maintain favorable oral health behaviors. These findings underline the need for preventive programs to address not only clinical and behavioral factors but also the emotional dynamics within families, integrating parental guidance and education as part of comprehensive oral health promotion [18]. The cognitive vulnerability model explains that children internalize parental anxieties through direct and indirect psychosocial interactions, with parental perceptions of threat and vulnerability mediating the relationship between negative dental experiences and child dental fear [19]. Meta-analytic evidence confirms that the association between parental and child dental fear is most pronounced in younger children, emphasizing the importance of early parent–child interactions and parental oral health behaviors in shaping children’s dental attitudes and anxiety [21]. Parental influence, whether positive or negative, affects not only the child’s behavior but also their perception of the environment. One Croatian study similarly reported a statistically significant correlation between children’s dental anxiety and that of their parents, supporting the cross-cultural relevance of these psychosocial mechanisms [26].

There are several limitations to this study. Although the participants were parents of young and preschool-aged children, the majority were mothers (over 97%), which limits the generalizability of the findings to fathers and male caregivers. Most respondents accessed the survey through social media, where parent groups are predominantly female, while father-only groups do not exist, limiting the diversity of participants. The survey distribution via social media also restricted access to individuals not active on these platforms. The questionnaire was closed-ended, which may have allowed for guessing, socially desirable responses, or inaccurate self-assessments. Additionally, the reliance on self-reported data may have led to an underestimation of anxiety-related behaviors or fear responses. Missing data were minimal and handled by listwise deletion, and duplicate responses were prevented through the survey design. No corrections for multiple comparisons were applied due to the exploratory nature of the analyses, which should be considered when interpreting the results. Future research could include longitudinal designs to evaluate the effects of educational interventions on both oral health knowledge and anxiety reduction, providing a more comprehensive understanding of behavioral changes over time.

## 5. Conclusions

This study highlights insufficient parental knowledge about oral health and non-adherence to guidelines regarding oral hygiene practices for their children. Further nationwide research is needed, including a larger sample size and a higher proportion of fathers, in order to raise awareness of the importance of oral health for children’s overall health and for a healthy society. Such research would also support the development of strategies for better promotion of oral health through informing, educating, and motivating parents as key figures in implementing measures to maintain and improve their children’s oral health. Improving oral health literacy should be complemented by strategies addressing parental fear and children’s dental trauma responses, as education alone may not fully mitigate avoidance and anxiety. Addressing these emotional and behavioral mechanisms is crucial for effective oral health promotion. Integrating psychological and communication-based interventions targeting parental dental anxiety provides a holistic approach, fostering positive oral health behaviors in children and aligning with contemporary psychosocial perspectives in pediatric dentistry and public health.

## Figures and Tables

**Table 1:** Sociodemographic data of the respondents (parents) in relation to the total knowledge score on oral health and caries risk factors.

Characteristic	Answer	N (%)	Oral Health Knowledge	*p*	Risk Factors Knowledge	*p*
Gender	Male	27 (2.8)	6.7 ± 3.0	0.002 *	12.9 ± 4.8	0.134
	Female	921 (97.2)	8.4 ± 2.4		14.2 ± 4.3	
Age	<29	175 (18.5)	8.3 ± 2.4	0.461	13.5 ± 5.0	0.099
	30–39	619 (65.3)	8.3 ± 2.4		14.3 ± 4.1	
	>40	154 (16.2)	8.4 ± 2.7		14.4 ± 4.0	
Education level	Elementary school	3 (0.3)	6.7 ± 1.5	0.320	13.7 ± 4.3	0.068
	High school	283 (29.9)	8.3 ± 2.5		14.2 ± 4.0	
	University education	662 (69.8)	8.4 ± 2.4		14.2 ± 4.3	
Employment status	Employed	832 (87.8)	8.0 ± 2.5	0.141	14.3 ± 4.3	0.094
	Unemployed	116 (12.2)	8.4 ± 2.4		13.3 ± 4.4	
Socioeconomic status	Below average	13 (1.4)	8.0 ± 2.4	0.021 *	13.2 ± 5.3	0.110
	Average	706 (74.5)	8.2 ± 2.5 ^a^		14.0 ± 4.4	
	Above average	229 (24.2)	8.8 ± 2.3 ^a^		14.8 ± 3.7	
Place of living	Rural	252 (26.6)	8.2 ± 2.5	0.353	13.6 ± 4.6	0.057
	Urban	696 (73.4)	8.4 ± 2.4		14.3 ± 4.2	
Career in healthcare	Yes	166 (17.5)	9.6 ± 2.3	≤0.001 *	15.6 ± 3.7	≤0.001 *
	No	782 (82.5)	8.1 ± 2.4		13.8 ± 4.3	
Number of children	1	471 (49.7)	8.2 ± 2.5 ^a^	0.010 *	14.3 ± 4.2	0.381
	2	335 (35.3)	8.6 ± 2.4 ^a^		14.0 ± 4.6	
	≥3	142 (15.0)	8.3 ± 2.5		14.0 ± 4.0	

Data are presented as the frequency and percentages, as well as mean values and standard deviation. * The data were tested using the Mann–Whitney U test or Kruskal–Wallis test (*p* < 0.05). The same superscript lowercase letters indicate a statistical difference between groups.

**Table 2:** Characteristics of the respondents’ children.

Characteristic	Answer	N (%)	Oral Health Knowledge	*p*	Risk Factors Knowledge	*p*
Gender	Male	462 (48.7)	8.3 ± 2.5	0.565	14.1 ± 4.5	0.676
	Female	486 (51.3)	8.3 ± 2.3		14.2 ± 4.1	
Age (years)	0–3	556 (58.6)	8.3 ± 2.4	0.669	14.5 ± 4.2	0.005 *
	4–7	392 (41.4)	8.4 ± 2.5		13.7 ± 4.3	
Age of first tooth eruption (months)	<6	290 (30.6)	8.2 ± 2.3	0.008 *	13.7 ± 4.4	0.053
	6–8	500 (52.7)	8.5 ± 2.5 ^a^		14.4 ± 4.2	
	>8	158 (16.7)	8.0 ± 2.5 ^a^		14.1 ± 4.2	

Data are presented as frequencies and percentages, as well as mean values and standard deviations. * The data were tested using the Mann–Whitney U test or Kruskal–Wallis test (*p* < 0.05). The same superscript lowercase letters indicate a statistical difference between groups.

**Table 3:** Distribution of responses to questions assessing parents’ knowledge about children’s oral health.

Question	Answer	N(%)
Mother’s oral flora is transmitted to the newborn, and a higher presence of cariogenic bacteria in the mother increases the chances of the child developing caries.	True	274 (28.9)
	False	674 (71.1)
Oral health is crucial for overall health.	True	864 (91.1)
	False	84 (8.9)
Dental caries is caused by bacteria.	True	678 (71.5)
	False	270 (28.5)
Adequate oral hygiene habits, including the use of fluoride, help protect teeth from caries.	True	775 (81.8)
	False	173 (18.2)
Although the first primary tooth can erupt between 4 and 10 months, the first tooth usually erupts around the age of 6 months.	True	739 (78.0)
	False	209 (22.0)
The first permanent teeth to erupt in the oral cavity between 6–7 years of age are the first permanent molars.	True	379 (40.0)
	False	569 (60.0)
On average, a child has 20 primary teeth by the age of three.	True	744 (78.5)
	False	204 (21.5)
Tooth brushing should begin at the eruption of the first primary tooth and should be performed at least twice daily.	True	854 (90.1)
	False	94 (9.9)
Parents should at least supervise and assist the child with brushing until the age of seven.	True	889 (93.8)
	False	59 (6.2)
From the eruption of the first tooth until the age of three, it is recommended to use fluoride toothpaste the size of a smear or a grain of rice twice daily.	True	614 (64.8)
	False	334 (35.2)
For children aged 3–6 years, it is recommended to use pea-sized fluoride toothpaste twice daily.	True	702 (74.1)
	False	246 (25.9)
Ideally, children should have their first orthodontic examination around the age of seven.	True	397 (41.9)
	False	551 (58.1)

Data are presented as frequencies and percentages.

**Table 4:** Respondents’ knowledge regarding the association of risk factors with early childhood caries.

Question	Answer	N(%)
Mother/primary caregiver has active dental caries	Yes	427 (45.0)
	No	521 (55.0)
Parents’ level of education and literacy	Yes	528 (55.7)
	No	420 (44.3)
Parents’ social status	Yes	332 (35.0)
	No	616 (65.0)
Parents’ knowledge about oral health	Yes	833 (87.9)
	No	115 (12.1)
Parents’ oral hygiene habits	Yes	850 (89.7)
	No	98 (10.3)
Psychosocial factors of parents	Yes	666 (70.3)
	No	282 (29.7)
Lack of time and presence of parents to perform oral hygiene	Yes	827 (87.2)
	No	121 (12.8)
Parents’ beliefs and attitudes about oral health	Yes	832 (87.8)
	No	116 (12.2)
Environmental factors (e.g., parental smoking)	Yes	454 (47.9)
	No	494 (52.1)
Child’s lack of cooperation and resistance to tooth brushing	Yes	695 (73.3)
	No	253 (26.7)
Lack of access to healthcare	Yes	580 (61.2)
	No	368 (38.8)
Financial difficulties	Yes	393 (41.5)
	No	555 (58.5)
Consumption of sugary drinks and snacks more than 3 times a day between meals	Yes	867 (91.5)
	No	81 (8.5)
Consumption of sugary drinks and food before bedtime	Yes	879 (92.7)
	No	69 (7.3)
Insufficiently developed enamel or deep fissures on teeth	Yes	539 (56.9)
	No	409 (43.1)
Food impaction between adjacent tooth surfaces	Yes	672 (70.9)
	No	276 (29.1)
Accumulation of dental plaque (deposits)	Yes	726 (76.6)
	No	222 (23.4)
Inadequate oral hygiene habits	Yes	870 (91.8)
	No	78 (8.2)
Not attending regular dental check-ups	Yes	849 (89.6)
	No	99 (10.4)
Not using fluoride and other remineralization products	Yes	591 (62.3)
	No	357 (37.7)

Data are presented as frequencies and percentages.

**Table 5:** Respondents’ habits in caring for their child’s oral health.

Question	Answer	N (%)
Visiting the dentist if the child has dental caries	Yes	890 (93.9)
	No	58 (6.1)
History of visiting the dentist	Yes	715 (75.4)
	No	233 (24.6)
Tooth brushing frequency ≥ 2 times/day	Yes	744 (78.5)
	No	204 (21.5)
Tooth brushing duration ≥ 2 min	Yes	619 (65.3)
	No	329 (34.7)
Dental check-ups ≥ 2 times/year	Yes	632 (66.7)
	No	316 (33.3)
Use of separate toothbrushes for parent and child	Yes	937 (98.8)
	No	11 (1.2)
Replacing the toothbrush every 3 months	Yes	862 (87.1)
	No	122 (12.9)
Use of fluoride toothpaste	Yes	651 (68.7)
	No	297 (31.3)
Use of dental floss	Yes	238 (25.1)
	No	710 (74.9)
The parent regularly/always reminds the child to rinse the mouth and brush teeth after eating	Yes	391 (41.2)
	No	557 (58.8)
Parent supervises and assists with tooth brushing	Yes	883 (93.1)
	No	65 (6.9)
Starting tooth brushing at the eruption of the first primary tooth	Yes	685 (72.3)
	No	263 (27.7)
First dental visit by 12 months of age	Yes	316 (33.3)
	No	632 (66.7)

Data are presented as frequencies and percentages.

**Table 6:** Experience of oral health problems in the respondents’ children.

Question	Answer	N (%)
Dental caries	Yes	211 (22.3)
	No	737 (77.7)
Toothache	Yes	93 (9.8)
	No	855 (90.2)
Swelling	Yes	39 (4.1)
	No	909 (95.9)
Tooth injury—trauma	Yes	98 (10.3)
	No	850 (89.7)
Gingival infection	Yes	19 (2.0)
	No	929 (98.0)
Tooth sensitivity	Yes	47 (5.0)
	No	901 (95.0)
Bruxism	Yes	147 (15.5)
	No	801 (84.5)
Bad breath	Yes	108 (11.4)
	No	840 (88.6)
Thumb sucking	Yes	68 (7.2)
	No	880 (92.8)
Incorrect tongue position	Yes	47 (5.0)
	No	901 (95.0)
Lip biting	Yes	22 (2.3)
	No	926 (97.7)
Malocclusion	Yes	48 (5.1)
	No	900 (94.9)
Oral mucosa ulcerations	Yes	28 (3.0)
	No	920 (97.0)
Oral candidiasis	Yes	24 (2.5)
	No	924 (97.5)

Data are presented as frequencies and percentages.

**Table 7:** Parents’ self-assessment and assessment of children’s health and hygiene habits.

Question	Answer	N (%)	Oral Health Knowledge	*p*	Risk Factors Knowledge	*p*
Parents’ general health	Poor	9 (0.9)	8.3 ± 2.5	0.233	13.0 ± 5.5	0.653
	Good	563 (59.4)	8.4 ± 2.4		14.2 ± 4.2	
	Excellent	376 (39.7)	8.2 ± 2.4		14.0 ± 4.4	
Parents’ oral health	Poor	65 (6.9)	8.9 ± 2.1 ^a^	0.011 *	14.9 ± 4.2	0.225
	Good	521 (54.9)	8.4 ± 2.5		14.1 ± 4.4	
	Excellent	362 (38.2)	8.1 ± 2.4 ^a^		14.0 ± 4.2	
Parents’ oral health knowledge	Poor	21 (2.2)	8.6 ± 2.9	0.686	14.0 ± 5.3	0.611
	Good	631 (66.6)	8.4 ± 2.4		14.2 ± 4.3	
	Excellent	296 (31.2)	8.3 ± 2.4		14.0 ± 4.2	
Parents’ oral hygiene practices	Poor	33 (3.5)	8.6 ± 2.2	0.422	15.1 ± 3.5	0.392
	Good	506 (53.4)	8.4 ± 2.4		14.0 ± 4.5	
	Excellent	409 (43.1)	8.2 ± 2.4		14.3 ± 4.0	
Child’s general health	Poor	2 (0.2)	10.5 ± 2.1 ^a^	0.048 *	4.0 ± 5.7	0.075
	Good	245 (25.8)	8.6 ± 2.3		14.3 ± 4.3	
	Excellent	701 (73.9)	8.2 ± 2.5 ^a^		14.1 ± 4.3	
Child’s oral health	Poor	19 (2.0)	8.7 ± 1.6	0.249	14.7 ± 3.4	0.770
	Good	345 (36.4)	8.5 ± 2.4		14.2 ± 4.4	
	Excellent	584 (61.6)	8.2 ± 2.4		14.1 ± 4.3	
Child’s oral hygiene practices	Poor	27 (2.8)	8.8 ± 1.9	0.777	13.9 ± 3.6	0.518
	Good	483 (50.9)	8.3 ± 2.4		14.2 ± 4.4	
	Excellent	438 (46.2)	8.3 ± 2.5		14.1 ± 4.2	

Data are presented as frequencies and percentages, as well as mean values and standard deviations. * The data were tested using the Mann–Whitney U test or Kruskal–Wallis test (*p* < 0.05). The same superscript lowercase letters indicate a statistical difference between groups.

## Data Availability

The data are available from the corresponding author upon reasonable request.

## References

[1] Anil S., Anand P.S. (2017). Early Childhood Caries: Prevalence, Risk Factors, and Prevention. Front. Pediatr..

[2] Colak H., Dülgergil C.T., Dalli M., Hamidi M.M. (2013). Early childhood caries update: A review of causes, diagnoses, and treatments. J. Nat. Sci. Biol. Med..

[3] Zou J., Du Q., Ge L., Wang J., Wang X., Li Y., Song G., Zhao W., Chen X., Jiang B. (2022). Expert consensus on early childhood caries management. Int. J. Oral Sci..

[4] Damle S.G., Yadav R., Garg S., Dhindsa A., Beniwal V., Loomba A., Chatterjee S. (2016). Transmission of mutans streptococci in mother-child pairs. Indian J. Med. Res..

[5] Blankson P.K., Amoah G., Thadani M., Newman-Nartey M., Amarquaye G., Hewlett S., Ampofo P., Sackeyfio J. (2022). Prevalence of oral conditions and associated factors among schoolchildren in Accra, Ghana: A cross-sectional study. Int. Dent. J..

[6] Muthu M.S., Vandana S., Akila G., Anusha M., Kandaswamy D., Aswath Narayanan M.B. (2024). Global variations in eruption chronology of primary teeth: A systematic review and meta-analysis. Arch. Oral Biol..

[7] Ramakrishnan M., Banu S., Ningthoujam S., Samuel V.A. (2019). Evaluation of knowledge and attitude of parents about the importance of maintaining primary dentition—A cross-sectional study. J. Fam. Med. Prim. Care.

[8] Wang L.J., Tang R., Bonstein T., Bush P., Nancollas G.H. (2006). Enamel demineralization in primary and permanent teeth. J. Dent. Res..

[9] Butera A., Maiorani C., Morandini A., Simonini M., Morittu S., Trombini J., Scribante A. (2022). Evaluation of Children Caries Risk Factors: A Narrative Review of Nutritional Aspects, Oral Hygiene Habits, and Bacterial Alterations. Children.

[10] Riggs E., Kilpatrick N., Slack-Smith L., Chadwick B., Yelland J., Muthu M.S., Gomersall J.C. (2019). Interventions with pregnant women, new mothers and other primary caregivers for preventing early childhood caries. Cochrane Database Syst. Rev..

[11] Kitsaras G., Goodwin M., Kelly M.P., Pretty I.A. (2021). Bedtime Oral Hygiene Behaviours, Dietary Habits and Children’s Dental Health. Children.

[12] Saheb S.A.K., Najmuddin M., Nakhran A.M., Mashhour N.M., Moafa M.I., Zangoti A.M. (2023). Parents’ Knowledge and Attitudes toward Preschool’s Oral Health and Early Childhood Caries. Int. J. Clin. Pediatr. Dent..

[13] Wright J.T., Hanson N., Ristic H., Whall C.W., Estrich C.G., Zentz R.R. (2014). Fluoride toothpaste efficacy and safety in children younger than 6 years: A systematic review. J. Am. Dent. Assoc..

[14] Manchanda S., Sardana D., Liu P., Lee G.H., Li K.Y., Lo E.C., Yiu C.K. (2022). Topical fluoride to prevent early childhood caries: Systematic review with network meta-analysis. J. Dent..

[15] Bulut G., Bulut H. (2020). Zero to five years: First dental visit. Eur. J. Paediatr. Dent..

[16] Bagattoni S., Nascimben F., Biondi E., Fitzgibbon R., Lardani L., Gatto M.R., Piana G., Mattarozzi K. (2022). Preparing Children for Their First Dental Visit: A Guide for Parents. Healthcare.

[17] Alwusaybie M.M., AlBaqshi F.S., Alshaks M.A., Alabdullah M.Z., Alwosaibei M.A., AlBahrani H.J., Alsaleh H.M., Almadeh N.M., Alyousef A.H. (2024). Parental Knowledge and Awareness Regarding Early Orthodontic Consultation in Children: A Cross-Sectional Study. Cureus.

[18] Bayón G., Stiernhufvud F., Ribas-Pérez D., Biedma Perea M., Mendoza Mendoza A. (2025). Parental Anxiety Disorders and Their Impact on Dental Treatment in Children Aged 4 to 13 Years: A Cross-Sectional Observational Study. J. Clin. Med..

[19] Crego A., Carrillo-Diaz M., Armfield J.M., Romero M. (2013). Applying the Cognitive Vulnerability Model to the analysis of cognitive and family influences on children’s dental fear. Eur. J. Oral Sci..

[20] Uziel N., Meyerson J., Kuskasy M., Gilon E., Eli I. (2023). The Influence of Family Milieu on Dental Anxiety in Adolescents—A Cross-Sectional Study. J. Clin. Med..

[21] Themessl-Huber M., Freeman R., Humphris G., MacGillivray S., Terzi N. (2010). Empirical evidence of the relationship between parental and child dental fear: A structured review and meta-analysis. Int. J. Paediatr. Dent..

[22] Gavic L., Tadin A., Mihanovic I., Gorseta K., Cigic L. (2018). The role of parental anxiety, depression, and psychological stress level on the development of early-childhood caries in children. Int. J. Paediatr. Dent..

[23] Radić M., Benjak T., Vukres V.D., Rotim Ž., Zore I.F. (2015). Presentation of DMFT/dmft Index in Croatia and Europe. Acta Stomatol. Croat..

[24] Lešić S., Dukić W., Šapro Kriste Z., Tomičić V., Kadić S. (2019). Caries prevalence among schoolchildren in urban and rural Croatia. Cent. Eur. J. Public Health.

[25] Reic T., Galic T., Milatic K., Negovetic Vranic D. (2019). Influence of nutritional and oral hygiene habits on oral health in Croatian island children of school age. Eur. J. Paediatr. Dent..

[26] Petrović D., Cicvarić O., Šimunović-Erpušina M., Ivančić Jokić N., Bakarčić D., Bučević Sojčić P., Jurić H. (2024). The Role of Family Factors in the Development of Dental Anxiety in Children. Medicina.

[27] Gavic L., Tadin A., Matkovic A., Gorseta K., Sidhu S.K. (2022). The association of parental dental anxiety and knowledge of caries preventive measures with psychological profiles and children’s oral health. Eur. J. Paediatr. Dent..

[28] Badrov M., Matijević M., Tadin A. (2025). Parental Knowledge of Breastfeeding and Nutrition: Influence on Oral Health and Self-Reported Early Childhood Caries in Preschool Children in Croatia. Pediatr. Rep..

[29] Ivanisevic Z., Uzarevic Z., Lesic S., Vcev A., Matijevic M. (2021). Oral Health of Children from the SOS Children’s Village in Croatia. Int. J. Environ. Res. Public Health.

[30] Gavic L., Brekalo M., Tadin A. (2024). Caregiver Perception of the Oral-Health-Related Quality of Life of Children with Special Needs: An Exploratory Study. Epidemiologia.

[31] Badrov M., Perkov L., Tadin A. (2025). The Impact of Oral Health on the Quality of Life of Children with Autism Spectrum Disorder and Their Families: Parental Perspectives from an Online Cross-Sectional Study. Oral.

[32] Bakarčić D., Vlah N., Cicvarić O., Petrović D., Šimunović-Erpušina M., Janković S., Dragaš Zubalj N., Kresina S., Mohorić S., Gržić R. (2025). An Oral Health Promotion Model Implemented in the Primorje-Gorski Kotar County. Medicina.

[33] Eysenbach G. (2004). Improving the quality of Web surveys: The Checklist for Reporting Results of Internet E-Surveys (CHERRIES). J. Med. Internet Res..

[34] Croatian Bureau of Statistics (2024). Natural Population Change in 2023.

[35] Raosoft, Inc (2004). Sample Size Calculator.

[36] Nassar A.A., Fatani B.A., Almobarak O.T., Alotaibi S.I., Alhazmi R.A., Marghalani A.A. (2022). Knowledge, Attitude, and Behavior of Parents Regarding Early Childhood Caries Prevention of Preschool Children in Western Region of Saudi Arabia: A Cross-Sectional Study. Dent. J..

[37] Wilson A., Brega A.G., Batliner T.S., Henderson W., Campagna E.J., Fehringer K., Gallegos J., Daniels D., Albino J. (2014). Assessment of parental oral health knowledge and behaviors among American Indians of a Northern Plains tribe. J. Public Health Dent..

[38] Al-Dahan H.M., Ismael S.A. (2023). Early childhood caries: Parents’ knowledge, attitude and practice towards its prevention in refugee camps in Erbil, Iraq. BMC Oral Health.

[39] Bloom B.S. (1968). Learning for mastery. Instruction and curriculum. Regional education laboratory for the Carolinas and Virginia. Eval. Comment.

[40] Chawłowska E., Karasiewicz M., Lipiak A., Cofta M., Fechner B., Lewicka-Rabska A., Pruciak A., Gerreth K. (2022). Exploring the Relationships between Children’s Oral Health and Parents’ Oral Health Knowledge, Literacy, Behaviours and Adherence to Recommendations: A Cross-Sectional Survey. Int. J. Environ. Res. Public Health.

[41] Sağlam C., Mojarrab N., Ahlat E.M., Şakı Çınarcık B., Ersin N., Ertuğrul F., Çoğulu D. (2025). Evaluation of the effectiveness of parental oral health education. J. Pediatr. Res..

[42] Abegaz N.T., Berhe H., Gebretekle G.B. (2019). Mothers/caregivers healthcare seeking behavior towards childhood illness in selected health centers in Addis Ababa, Ethiopia: A facility-based cross-sectional study. BMC Pediatr..

[43] Tadin A., Poljak Guberina R., Domazet J., Gavic L. (2022). Oral Hygiene Practices and Oral Health Knowledge among Students in Split, Croatia. Healthcare.

[44] Alqahtani A.M., Thirunavukkarasu A. (2024). Assessment of patient knowledge and perceptions towards orthodontic treatment in the Aljouf Region, Saudi Arabia: A cross-sectional study. PeerJ.

[45] Nepaul P., Mahomed O. (2020). Influence of Parents’ Oral Health Knowledge and Attitudes on Oral Health Practices of Children (5–12 Years) in a Rural School in KwaZulu-Natal, South Africa. J. Int. Soc. Prev. Community Dent..

[46] Vasireddy D., Sathiyakumar T., Mondal S., Sur S. (2021). Socioeconomic Factors Associated with the Risk and Prevalence of Dental Caries and Dental Treatment Trends in Children: A Cross-Sectional Analysis of National Survey of Children’s Health (NSCH) Data, 2016–2019. Cureus.

[47] Padung N., Singh S., Awasthi N. (2022). First Dental Visit: Age Reasons Oral Health Status and Dental Treatment Needs among Children Aged 1 Month to 14 Years. Int. J. Clin. Pediatr. Dent..

[48] Davidson K.W., Barry M.J., Mangione C.M., Cabana M., Caughey A.B., Davis E.M., Donahue K.E., Doubeni C.A., Kubik M., US Preventive Services Task Force (2021). Screening and Interventions to Prevent Dental Caries in Children Younger Than 5 Years: US Preventive Services Task Force Recommendation Statement. JAMA.

[49] Jokić N.I., Bakarčić D., Janković S., Malatestinić G., Dabo J., Majstorović M., Vuksan V. (2013). Dental caries experience in Croatian school children in Primorsko-Goranska county: A Pilot Study. Cent. Eur. J. Public Health.

